# Supplementary material for: Splice-Junction-Based Mapping of Alternative Isoforms in the Human Proteome
Source: Cell Rep. Author manuscript; Available in PMC 2020 Jan 15. (PMC6961840; doi:10.1016/j.celrep.2019.11.026)
Supplement: 2 [file NIHMS1546469-supplement-2.zip › DF1/PXD010154/Heart/Heart_55_NID1_TCYDVDECQPSR.pdf]

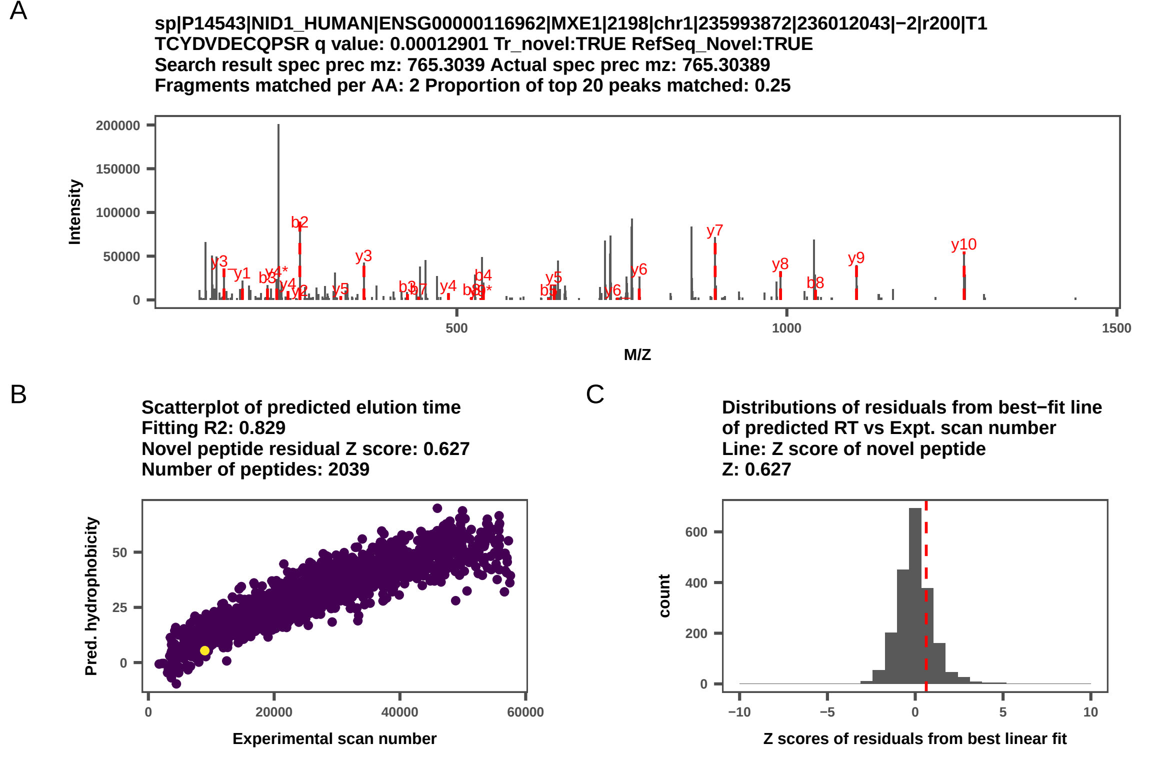
A
sp|P14543|NID1_HUMAN|ENSG00000116962|MXE1|2198|chr1|235993872|236012043|−2|r200|T1
TCYDVDECQPSR q value: 0.00012901 Tr_novel:TRUE RefSeq_Novel:TRUE
Search result spec prec mz: 765.3039 Actual spec prec mz: 765.30389
Fragments matched per AA: 2 Proportion of top 20 peaks matched: 0.25
Intensity
200000
150000
100000
50000
0
b2
y3
y7
y10
y9
y8
b8
y6
y5
b4
y1
y3
b3
y4*
y4
b5
y6
500
1000
1500
M/Z
B
Scatterplot of predicted elution time
Fitting R2: 0.829
Novel peptide residual Z score: 0.627
Number of peptides: 2039
Pred. hydrophobicity
50
25
0
0
20000
40000
60000
Experimental scan number
C
Distributions of residuals from best−fit line
of predicted RT vs Expt. scan number
Line: Z score of novel peptide
Z: 0.627
count
600
400
200
0
−10
−5
0
5
10
Z scores of residuals from best linear fit
